# Cyclin-dependent kinase 5-mediated phosphorylation of chloride intracellular channel 4 promotes oxidative stress-induced neuronal death

**DOI:** 10.1038/s41419-018-0983-1

**Published:** 2018-09-20

**Authors:** Dong Guo, Wenting Xie, Pan Xiong, Huifang Li, Siqi Wang, Guimiao Chen, Yuehong Gao, Jiechao Zhou, Ye Zhang, Guojun Bu, Maoqiang Xue, Jie Zhang

**Affiliations:** 1 Fujian Provincial Key Laboratory of Neurodegenerative Disease and Aging Research, Institute of Neuroscience, Medical College, Xiamen University, 361005 Xiamen, Fujian China; 2 The Institute of Cell Metabolism, Shanghai Key Laboratory of Pancreatic Disease, Shanghai General Hospital, School of Medicine, Shanghai Jiaotong University, 201620 Shanghai, China; 3 Department of Basic Medical Sciences, Medical College, Xiamen University, 361005 Xiamen, Fujian China

## Abstract

Oxidative stress can cause apoptosis in neurons and may result in neurodegenerative diseases. However, the signaling mechanisms leading to oxidative stress–induced neuronal apoptosis are not fully understood. Oxidative stress stimulates aberrant activation of cyclin-dependent kinase 5 (CDK5), thought to promote neuronal apoptosis by phosphorylating many cell death-related substrates. Here, using protein pulldown methods, immunofluorescence experiments and in vitro kinase assays, we identified chloride intracellular channel 4 (CLIC4), the expression of which increases during neuronal apoptosis, as a CDK5 substrate. We found that activated CDK5 phosphorylated serine 108 in CLIC4, increasing CLIC4 protein stability, and accumulation. Pharmacological inhibition or shRNA-mediated silencing of CDK5 decreased CLIC4 levels in neurons. Moreover, CLIC4 overexpression led to neuronal apoptosis, whereas knockdown or pharmacological inhibition of CLIC4 attenuated H_2_O_2_-induced neuronal apoptosis. These results implied that CLIC4, by acting as a substrate of CDK5, mediated neuronal apoptosis induced by aberrant CDK5 activation. Targeting CLIC4 in neurons may therefore provide a therapeutic approach for managing progressive neurodegenerative diseases that arise from neuronal apoptosis.

## Introduction

Oxidative stress is believed to be mediated by excessive exposure of cells to reactive oxygen species (ROS), like hydrogen peroxide (H_2_O_2_), and is often etiologically linked with cell apoptosis via different pathways, such as increase in intracellular Ca^2+^ concentration, mitochondrial dysfunction, and DNA damage^1,2^. Due to the high rate of oxidative metabolism and low level of antioxidant enzymes in the brain, neuronal cells are more vulnerable to oxidative stress^3^. These factors make oxidative stress as a critical pathogenic factor for neurodegenerative diseases, like Alzheimer’s disease (AD), which is featured with massive neuronal apoptosis^4^.

Cyclin-dependent kinase 5 (CDK5) is a member of the highly conserved cyclin-dependent kinase family, but it has not been found to be functional in promoting cell cycle progression as other traditional members^5^. Although CDK5 is ubiquitously expressed, its activators p35 and p39 are neuronal-specific proteins, which confine CDK5 kinase activity prominently in the nervous system. Physiologically, p35 binds CDK5 to endow activity with CDK5 in neuronal migration, differentiation, and maturation^6^. p35 locates at the cell membrane through myristoylation. Under neuronal toxic stress conditions, like oxidative stress, p35 could be cleaved by calpain and generate p25. As a fragment of p35,p25 loses the myristoylation site and is resistant to ubiquitin-mediated proteolysis^7^. CDK5/p25 exhibits extended activity and altered substrate specificity. CDK5/p25 phosphorylates a number of substrates that have important roles in neuronal apoptosis, including tau^8^, MEF2^9^, and FOXO1^10^, etc.

Chloride intracellular channel 4 (CLIC4) is one member of the CLIC protein family. CLIC4 expresses in various tissues including brain. As a chloride channel, CLIC4 locates at the plasma membrane, mitochondria, endoplasmic reticulum and other organelle membrane in an “unfolded membrane-inserted state”. CLIC4 also exists in a “folded globular state” in the cytoplasm and nucleus, in which the function of CLIC4 has not been well illuminated^11^. CLIC4 is involved in multiple cellular processes including protein trafficking^12^, cell adhesion and differentiation^13^, immune response^14^, and apoptosis^15^. Accumulating evidences indicate that CLIC4 protein levels were increased in apoptotic cells, including keratinocytes, β-cells, and glioma cells^16,17^. While the mRNA levels of CLIC4 were reported unchanged during cell apoptosis^16^. Upregulation of CLIC4 was also paralleled with the increase of Bax/Bcl-2 ratio, cytochrome *C* releasing, and cleaved caspase3^17^. The nuclear translocation of CLIC4 is also reported during certain cell apoptosis^18^. Although CLIC4 is highly involved in cell apoptosis, the regulation of CLIC4 in neuronal apoptosis remains to be explored.

Here, we reported marked upregulation of CLIC4 protein level during neuronal apoptosis. The aberrant activation of CDK5 mediates the phosphorylation of CLIC4 on Ser108, increasing CLIC4 protein stability and leading to CLIC4 protein accumulation. CLIC4 acts as a downstream effector of CDK5 to mediate cell apoptosis in several neuronal death models in vitro and in vivo. CLIC4 inhibitor attenuates neuronal death mediated by activation of CDK5. Our study is beneficial to the understanding of the neuronal toxicity of CDK5 and suggests that CLIC4 may be a potential therapeutic target for neurological diseases with oxidative stress-induced neuronal death.

## Materials and methods

### Animals

All experimental procedures involved were performed according to protocols approved by the Institutional Animal Care and Use Committee at Xiamen University. C57BL/6 mice at 6 to 8 weeks of age from the Xiamen University Laboratory Animal Center were used. A colony of Cdk5−/+ mice from the Jackson Laboratory (Bar Harbor, ME) were maintained on a mixed C57BL/6Jx129/S1 background. Homozygous mutant embryos were produced by intercrossing heterozygous Cdk5−/+ mice and genotyped as described previously^19^.

### Reagents

All chemicals were purchased from Sigma-Aldrich unless noted otherwise. Antibodies against c-myc, cleaved caspase3, cleaved PARP, and GST were purchased from Cell Signaling Technology (Beverly, MA). Anti-GAPDH, γ-H2A.X, β-actin, CDK5, p35, GFP, HA, p-S/T/Y, and Thiophosphate ester antibodies were from Abcam (Cambridge, MA). Anti-CLIC4 antibody was from Novagen (Madison, WI). Fluorescent anti-mouse or anti-rabbit IgG antibodies conjugated with Alexa Fluro 488 or Alexa Fluro 594 were from Invitrogen. Protein G Dynabeads were from Life technologies, and Glutathione Sepharose beads were from GE. ATP-γ-S and *p*-nitrobenzyl mesylate (PNBM) were from Abcam.

### Plasmid constructs

All restriction enzymes and T4 ligases were purchased from Thermo Scientific (San Diego, CA). CLIC4 was ligated into pCMV-HA, pcDNA3.1/myc-His, pRK5-mGST, and pCAG-IRES-EGFP vectors. CDK5, p35, and p25 were ligated into pCAG-IRES-EGFP, pRK5-mGST, and pET-his vectors. GFP-tagged CDK5, CDK5-KD (D144N), and p25 were constructed with pEGFP-C3 vector. Plasmids used in BiFC were prepared by ligating CDK5 and CLIC4 into VN173 and VC155, separately. CDK5 shRNA plasmid was prepared by ligating the nucleotides (TTGTCAGGCTTCATGATGTTT) into PLL3.7 vector. CLIC4 shRNA plasmids were constructed by ligating the nucleotides (a, TGGCAATGAAATGACATTA; b, CACCATTTATAACTTTCAA) into GV118 vector from GeneChem (Shanghai, China). All constructs were verified by sequencing.

### Cell culture

N2a cells were cultured in Dulbecco’s modified Eagle’s medium (GIBCO) supplemented with 10% fetal bovine serum (GIBCO), 100 units/ml penicillin, and 100 g/ml streptomycin in humidified, 37 °C chambers with 5% CO2. Primary cortical neurons were isolated from E16.5 embryos after being digested with 0.25% Trypsin-EDTA, and cultured on poly-lysine coated plates or coverslips in Neurobasal basic medium supplemented with 10% B27 supplements (GIBCO). Primary cerebellum granule neurons (CGNs) were isolated as the same way described above from D7 post-natal mouse cerebellum.

### Protein extraction, co-immunoprecipitation, GST pulldown, and western blotting

Cell pellets or tissues were homogenated with TNEN buffer (50 mM Tris-HCl 8.0, 150 mM NaCl, 5 mM EDTA and 1% NP-40) with protease inhibitors (Roche, USA) and phosphatase inhibitors (Roche, USA) and centrifuged at 14,000 × *g* for 10 min at 4 °C. For co-immunoprecipitation, the supernatant was mixed indicated antibodies overnight at 4 °C and then with 20 μl Protein G Dynabeads (Life technologies, Carlsbad, CA) for 2 h at 4 °C. For GST pulldown, GST, or GST-tagged proteins were precipitated for 2 h at 4 °C with Glutathione Sepharose (GE, Milwaukee, WI), and eluted with 10 mM Glutathione reduced solution following three times washing with TNEN buffer. Proteins were separated by SDS-PAGE gel and transferred to PVDF membranes. After blocking, membranes were probed with various primary antibodies and then HRP-linked secondary antibodies. Signals were detected using the ECL method.

### Immunocytochemistry

Primary neurons or HT22 cells grown on glass coverslips were washed with cold PBS and fixed with 4% paraformaldehyde for 15 min. Cells were then washed and permeabilized with 0.3% Triton X-100 for 10 min. After blocking with 5% BSA for 1 h, cells were probed with indicated antibodies overnight at 4 °C. Coverslips were washed with PBS, and secondary antibodies with specific fluorescence dyes were added and incubated for 1 h at room temperature. After being washed with PBS, coverslips were mounted to glass slides with ProLong Gold Antifade Reagent with DAPI (Invitrogen). Images were taken by fluorescence microscopy (Olympus Confocal Ti system).

### In vitro kinase assay

Recombined 6× His-tagged CDK5/p25 kinase complex was expressed in insect cells and purified via affinity chromatography and gel filtration. Recombinant GST-CLIC4 and GST-CLIC4 (S108A) protein, as well as GST tag protein, were purified from *E. coli* by affinity chromatography. Kinase assay was performed as described^20^. Briefly, about 100 ng substrate was added into kinase assay buffer (CST) containing 25 mM Tris-HCl (pH 7.5), 5 mM beta-glycerophosphate, 2 mM dithiothreitol (DTT), 0.1 mM Na_3_VO_4_, and 10 mM MgCl_2_, then incubated with 10 ng CDK5/p25 kinase and 50 μM ATP-γ-S at 30 °C for 45 min. The samples were then alkylated by 50 mM PNBM/5% DMSO, incubated for 1 h at room temperature, then subjected to SDS-PAGE and western blotting.

### Stereotactic injection

Wild-type adult C57BL/6 mice were first anesthetized and placed on a stereotaxic apparatus (RWD Life Science, Shanghai, China), then drug was injected into the right lateral ventricle (anteroposterior, −1.0 mm; mediolateral, −0.5 mm, dorsoventral, −2.0 mm) in a duration of 5 min. After injection, the needle was left in place for 10 min before being withdrawn to reduce backflow. Mice were allowed to recover in a dark warm place.

### In utero electroporation

A 2 μl of plasmid mix containing FastGreen dye (Thermo Scientific) was injected and electroporated into the lateral ventricle of E14.5 embryos using the ElectroSquarePorator ECM (BTX Genetronics), present at 50 ms pulses of 40 V with 950 ms intervals. Two days after electroporation, embryonic brains were dissected and fixed overnight in 4% PFA at 4 °C.

### Immunohistochemistry

Immunohistochemistry on frozen sections was carried out using the Ultrasensitive Immunohistochemistry Kit (MXB Biotechnologies, Fuzhou, China) following the manufacturer’s instructions. Briefly, fixed and dehydrated brain tissues were embed in embedding medium and sectioned to 10 μm slices. Tissue slides were rinsed with PBS and incubated with 0.3% H_2_O_2_ solution for 5 min to block endogenous peroxidase activity. Blocking buffer (10% goat serum) was added onto sections for 10 min at room temperature. Then primary antibodies were applied to the sections at 4 °C overnight. Secondary antibody was applied at room temperature for 30 min after PBS rinsing. Sections were rinsed with PBS again and fresh made DAB substrate solution was incubated until the desired color intensity is reached. Hematoxylin solution was added onto the sections for 2 min to stain the nucleus. Slides were dehydrated through four changes of alcohol (95, 95, 100 and 100%) and cleaned in xylene, then mounted with coverslips and neutral resins.

### Cell viability assay

As for the Trypan blue staining, after being exposed to drugs for the indicated time, cells were immediately stained with 1.5% Trypan blue (in PBS) for 10 min, and then fixed with 4% paraformaldehyde for 15 min. After being rinsed with PBS, cells were photographed under a Nikon eclipse Ti microscope. The viability of cells was determined by the percentage of the ratio of the unstained cell number to the total cell number. About 100 cells per coverslip were counted and at least three coverslips were used each group. As for the CCK-8 assay, the Cell Counting Kit (Dojindo, Kumamoto, Japan) was used according to its manual. Briefly, after drug exposure, cells in 100 μl culture medium in the 96-well plate were incubated with 10 μl CCK-8 solution at 37 °C for 1 h. Then reaction was stopped by adding 10 μl CCK-8 stop solution. Absorbance values at 450 nm were measured in a Thermo Scientific varioskan flash spectrophotometer.

### Statistical analysis

Immunoblotting signals were quantified by ImageJ software. Statistical analysis was performed with GraphPad Prism 6 (GraphPad Software Inc., La Jolla, CA) by unpaired two-tailed Student’s *t*-test or analysis of variance (ANOVA) test followed by a post hoc test. Significance values are indicated by asterisks: *P < 0.05; **P < 0.01; ***P < 0.001.

## Results

### CDK5 associates with CLIC4 and phosphorylates CLIC4 at serine 108

As a chloride channel protein, CLIC4 was reported to be upregulated during apoptosis of certain cells^16,17^. However, the regulation of CLIC4 in neuronal apoptosis still remains to be explored. Aberrant activation of CDK5 has been linked to the death of neurons induced by oxidative stress^21^. We hypothesized that CLIC4 may be involved in CDK5-mediated neuronal death. We started our analyses by investigating the physical association between CDK5 and CLIC4.

**Fig. 1:**
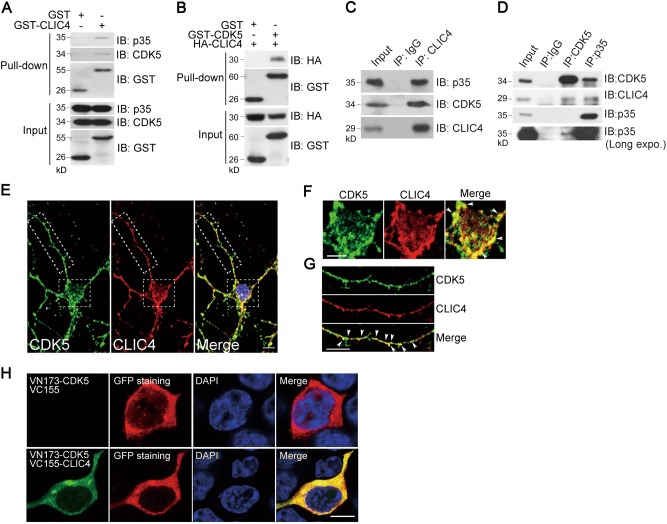
CLIC4 interacts with CDK5/p35. **a** Immunoblotting of proteins pulled down by glutathione Sepharose from lysates of N2a cells transiently transfected with GST alone or GST-CLIC4 as indicated. Membranes were probed with antibodies against the indicated proteins. **b** Immunoblotting of proteins pulled down by glutathione Sepharose from lysates of N2a cells transiently transfected with HA-CLIC4 and GST alone or GST-CLIC4. Membranes were probed with antibodies to the indicated proteins. **c** Immunoblotting of proteins immunoprecipitated from brain homogenates of wild-type C57BL/6 mice with antibodies against normal IgG or anti-CLIC4 antibody. **d** Immunoblotting of proteins immunoprecipitated from brain homogenates of wild-type C57BL/6 mice with antibodies against normal IgG, anti-CDK5 or anti-p35 antibody. **e**–**g** Immunofluorescence staining of DIV7 primary cortical neurons with anti-CDK5 and anti-CLIC4 antibodies showed the co-localization of CDK5 and CLIC4. Higher magnification around neuronal soma (**f**) and neurite (**g**) of the indicated area of **e** was presented and the co-localization was pointed with arrows. DAPI is a nucleus dye. Scale bar = 10 μm. **h** BiFC fluorescence and immunostaining showed that CDK5 and CLIC4 interacted in the cytoplasm of N2a cells. N2a cells were transfected with VN173-CDK5 and VC155 vector or VC155-CLIC4, then immunostained with anti-GFP antibody. DAPI is a nucleus dye. Scale bar = 10 μm

We found that glutathione *S*-transferase (GST) attached CLIC4, but not GST alone, pulled down CDK5 and p35 from N2a cell lysates (Fig. 1a). Similarly, over-expressed CLIC4 (HA-CLIC4) was pulled down with CDK5, when GST-CDK5 and HA-CLIC4 were co-expressed in N2a cells (Fig. 1b). Furthermore, CLIC4 was co-immunoprecipitated with CDK5 or p35 from mouse brain lysates (Fig. 1c, d), indicating that CLIC4 associates with CDK5 and p35 in the brain.

Consistent with these findings, immunofluorescence studies of cultured primary neurons revealed that CLIC4 is co-localized with CDK5 in the cytoplasm (Fig. 1e), the neuronal soma (Fig. 1f) and neurites (Fig. 1g). We used bimolecular fluorescence complementation (BiFC)^22^ to further confirm their interaction by co-expressing VN173-CDK5 and VC155-CLIC4 in N2a cells. If CDK5 interacts with CLIC4, the conjoint VN173 and VC155 could bind together as a full GFP (Venus-type) protein. Reconstituted GFP fluorescence produced by CDK5:CLIC4 binding appeared to be strong in the cytoplasm. Co-expression of VN173 control vector and VC155-CLIC4 were used as a negative control (Fig. 1h).

**Fig. 2:**
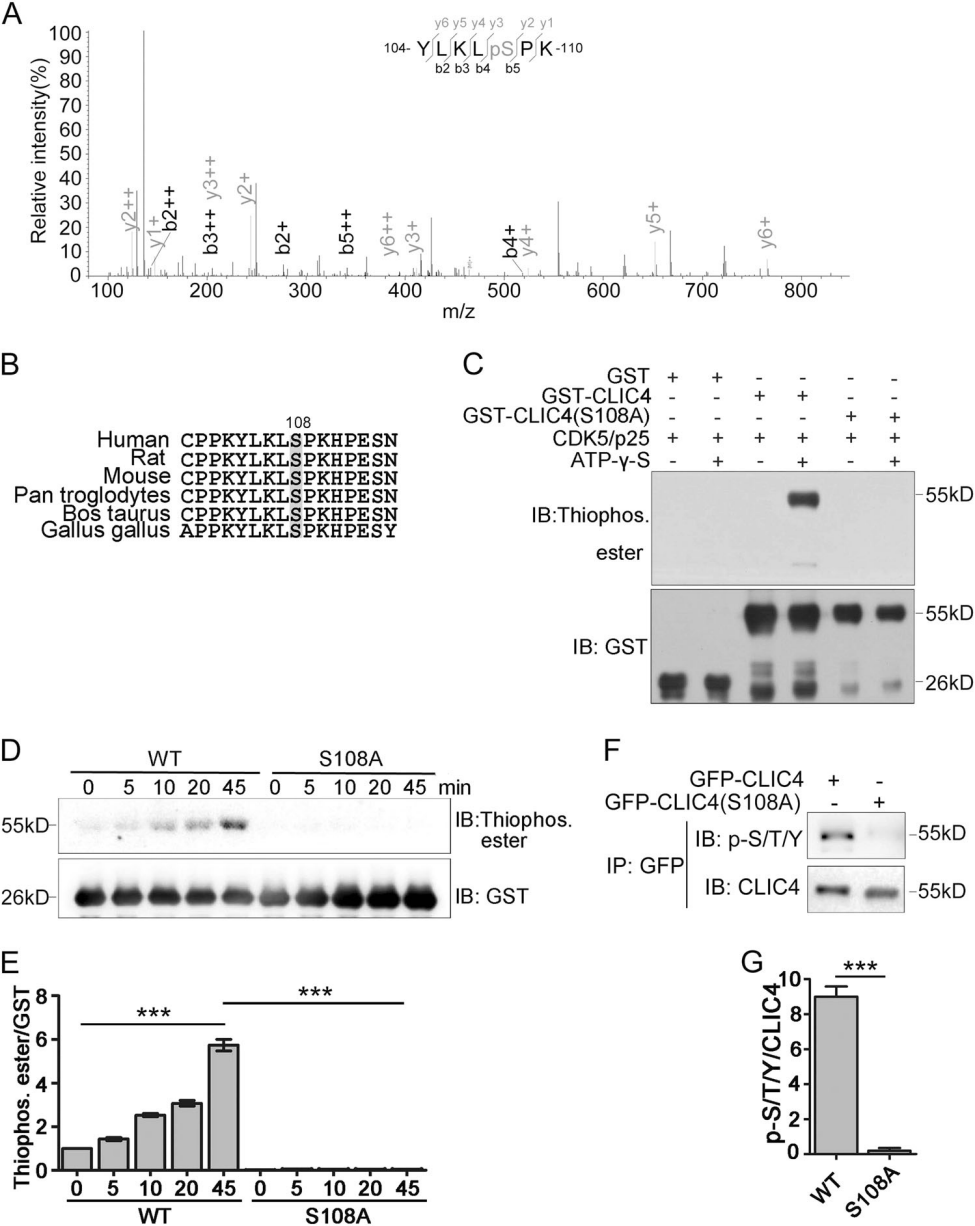
CDK5 phosphorylates CLIC4 at serine 108. **a** Purified GST-CLIC4 was phosphorylated by purified His-CDK5/p25 in vitro and analyzed by mass spectrometry. Mass spectrometric analysis was performed of a tryptic fragment matched to the +2 charged peptide 104-YLKLSPL-110; the results suggested that S108 was phosphorylated. The Mascot score was 27.5, and the expectation value was 1.39e−01. **b** Serine 108 and CDK5 phosphorylation consensus motif of CLIC4 are conserved in different species. **c** Immunoblotting of in vitro kinase assay performed by adding GST tag and recombined GST-CLIC4 or GST-CLIC4 (S108A) proteins into kinase assay buffer containing His-CDK5/p25 and ATP-γ-S. Anti-Thiophosphate ester antibody was used in western blotting to indicate the phosphorylated proteins. Anti-GST antibody was used to indicate the total proteins. **d**, **e** In vitro kinase assay in time course performed as described in **c** and reacted for different times as indicated. Thiophosphate ester/GST was quantified in **e** (*n* = 3 experiments). **f**, **g** Global phosphorylation levels of GFP-CLIC4 and GFP-CLIC4 (S108A) in cells. GFP-tagged proteins were precipitated with anti-GFP antibody from lysates of N2a cells transiently transfected with GFP-CLIC4 or GFP-CLIC4 (S108A). Samples were subjected to immunoblotting for p-S/T/Y and CLIC4. P-S/T/Y/CLIC4 was quantified in **g** (*n* = 3 experiments). Data are presented as the mean and SEM, and were analyzed by two-way ANOVA test (**e**) or unpaired Student’s *t*-test (**g**). *P < 0.05; **P < 0.01; ***P < 0.001

We then explored whether CDK5 can phosphorylate CLIC4. The recombined CLIC4 protein and active CDK5/p25 were co-incubated by in vitro kinase assay, and the samples were subjected to mass spectrometry to identify the phosphorylation modification of CLIC4. We identified that serine 108 of CLIC4 was phosphorylated in the presence of CDK5/p25 (Fig. 2a). CDK5 can phosphorylate serine or threonine in the motif S/TPXK/R/H where S and T are serine and threonine residents that can be phosphorylated (X is any amino acid and P is the obligatory proline presents at position + 1)^23^. According to CLIC4 protein sequence, serine 108 is in a classic CDK5 phosphorylation motif site (SPKH), which is also conserved in different species (Fig. 2b). To confirm the phosphorylation site, we mutated serine 108 to alanine (CLIC4-S108A). Utilizing in vitro kinase assay and Thiophosphate ester antibody test^20^, we observed that active CDK5/p25 complex is capable of phosphorylating purified GST attached CLIC4, but not CLIC4 (S108A) (Fig. 2c). These results confirm that CDK5 phosphorylates CLIC4 at serine 108. In vitro kinase assay was also performed in time course reactions. Phosphorylation level of wild-type CLIC4 was gradually increased with longer reaction time, while phosphorylation signal was not detectable when CLIC4 (S108A) was used (Fig. 2d, e).

Furthermore, we used an anti-p-S/T/Y antibody which recognizes phosphorylated serine/threonine/tyrosine to test the phosphorylation of CLIC4 in N2a cells. N2a cells were transfected with GFP-CLIC4 or GFP-CLIC4 (S108A). Then, GFP-CLIC4 or GFP-CLIC4 (S108A) was precipitated with anti-GFP antibody and subjected to western blotting with anti-p-S/T/Y antibody. The phosphorylation signal can only be detected with CLIC4 but not CLIC4 (S108A) (Fig. 2f, g).

### CLIC4 is accumulated in apoptotic neurons

**Fig. 3:**
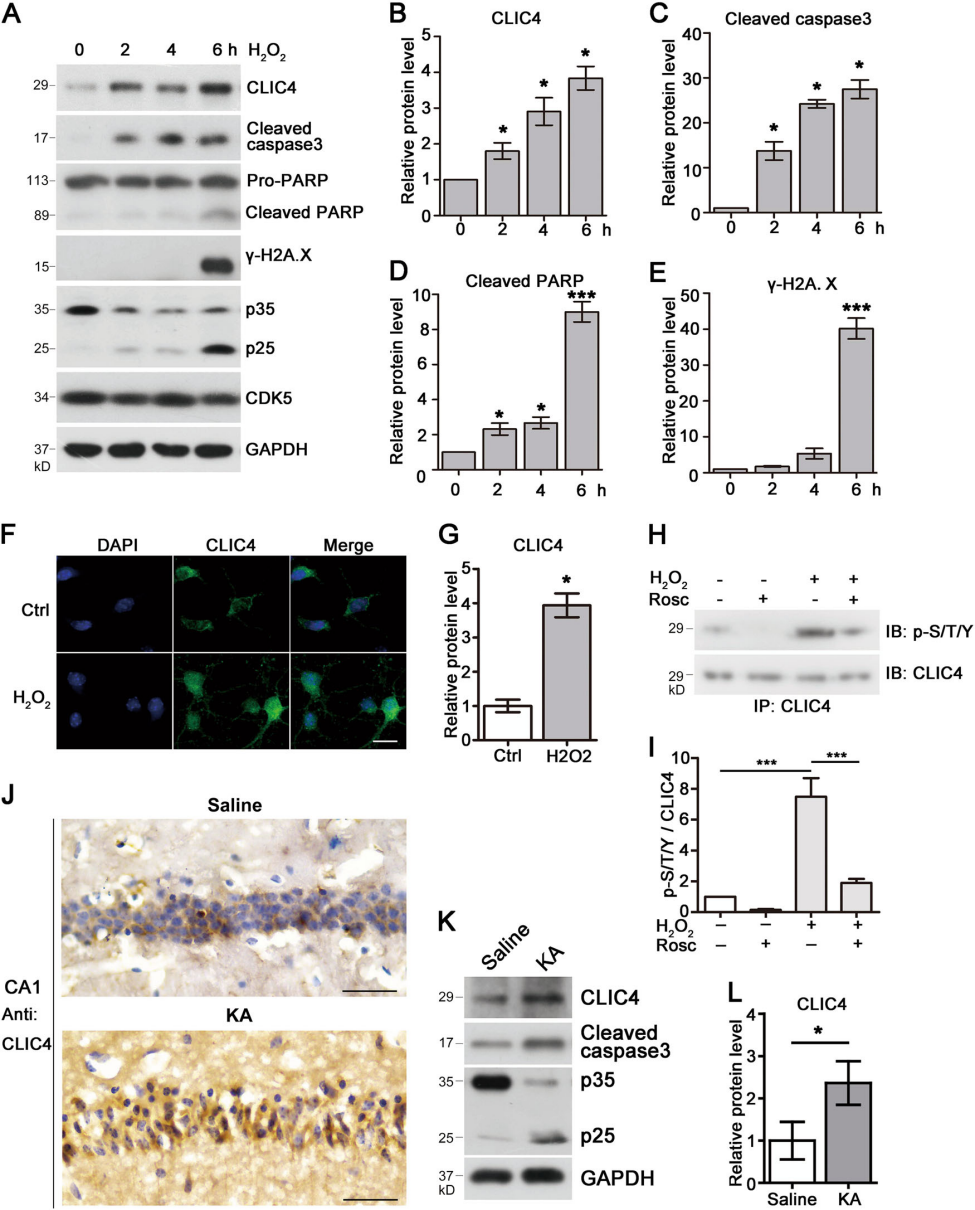
Increased protein level of CLIC4 in apoptotic neurons. **a**–**e** Immunoblotting of primary cortical neurons at DIV8 treated with 300 μM H_2_O_2_ for indicated times. Normalized CLIC4 (**b**), cleaved caspase3 (**c**), cleaved PARP (**d**), and γ-H2A.X (**e**) were quantified (*n* = 3 experiments). **f**, **g** Immunostaining of CLIC4 in primary cortical neurons treated with 300 μM H_2_O_2_ for 6 h. DAPI was used as a nucleus dye. The relative intensity of CLIC4 in each cell was quantified in G (*n* = 3 experiments, over 30 cells were quantified in each group). Scale bar = 20 μm. **h**, **i** Global phosphorylation levels of primary cortical neurons treated with 300 μM H_2_O_2_ for 1 h with or without 10 μM Roscovitine pre-treatment for 6 h. Endogenous CLIC4 was precipitated with anti-CLIC4 antibody, and samples were subjected to immunoblotting for CLIC4 and p-S/T/Y. p-S/T/Y/CLIC4 was quantified in **i** (*n* = 3 experiments). **j** CLIC4 immunostaining in the CA1 region of hippocampus from mouse injected with KA or saline. Slices were immunostained with anti-CLIC4 antibody by IHC. Scale bar = 100 μm. **k**, **l** Protein levels of CLIC4, p35/p25, and cleaved caspase3 in hippocampi of mice injected with KA or saline control. Relative CLIC4 levels were quantified in **l** (*n* = 3 experiments). Data are presented as the mean and SEM, and were analyzed by one-way ANOVA test followed by Dunnett test (**b**, **c**, **d**, **e**) or Tukey test (**i**) or unpaired Student’s *t*-test (**g**, **l**). *P < 0.05; **P < 0.01; ***P < 0.001

Previous reports showed that CLIC4 was upregulated and could induce cell apoptosis under different stress conditions in various cell types^16,17^. Nevertheless, whether CLIC4 is involved in neuronal death remains unknown. To test this, we used H_2_O_2_, an oxidative stress inducer that can cause neuronal apoptosis, to treat primary neurons. H_2_O_2_ treatment resulted in upregulation of CLIC4 tested by western blotting and immunostaining (Fig. 3a–g). The increased levels of cleaved caspase3, cleaved PARP and γ-H2A.X indicated that the neurons were undergoing apoptosis. Simultaneously, accumulation of p25 was also observed. Oxidative stress induced by H_2_O_2_ treatment can lead to aberrant activation of CDK5 by inducing the cleavage of p35 to p25^21^. To confirm that CLIC4 was also phosphorylated during neuronal death accompanied with CDK5 aberrant activation, endogenous CLIC4 was precipitated from neurons treated with H_2_O_2_ in the presence or not of CDK5 kinase inhibitor Roscovitine. Western blotting with p-S/T/Y antibody showed that the global phosphorylation level of CLIC4 was increased significantly under H_2_O_2_ treatment, while pre-treatment with Roscovitine repressed the CLIC4 phosphorylation (Fig. 3h, i). Staurosporine (STS) treatment could cause cell death in different cells including the neurons. We found that STS also increased CLIC4 protein levels when neurons were undergoing apoptosis accompany with p25 generation (Supp. Figure 1A–C). Cleavage of p35 to p25 is mediated by Ca^2+^-dependent protease calpain^24^. KCl withdrawal treatment was widely used to induce cerebellar granular neurons (CGNs) apoptosis by upregulating Ca^2+^ influx. CGNs treated with KCl withdrawal exhibited enhanced cleaved caspase3 levels and generation of p25 from p35. Notably, CLIC4 was markedly increased in CGNs with KCl withdrawal treatment measured by western blotting and immunostaining (Supp. Figure 1D–G).

To expand the finding that CLIC4 is also upregulated during neuronal death induced by other stimulation in vivo, kainic acid (KA) was injected into mouse brain to induce hippocampal neuronal death. We observed substantial increasing of CLIC4 protein levels in hippocampi from KA treated mice compared with saline controls (Fig. 3j–l). KA treatment also enhanced cleaved caspase3 and p25 levels, which indicates that CDK5 was aberrantly active. These results demonstrate that CLIC4 is increased in the process of neuronal apoptosis induced by various stimuli.

### CDK5-dependent phosphorylation of CLIC4 mediates its stabilization

**Fig. 4:**
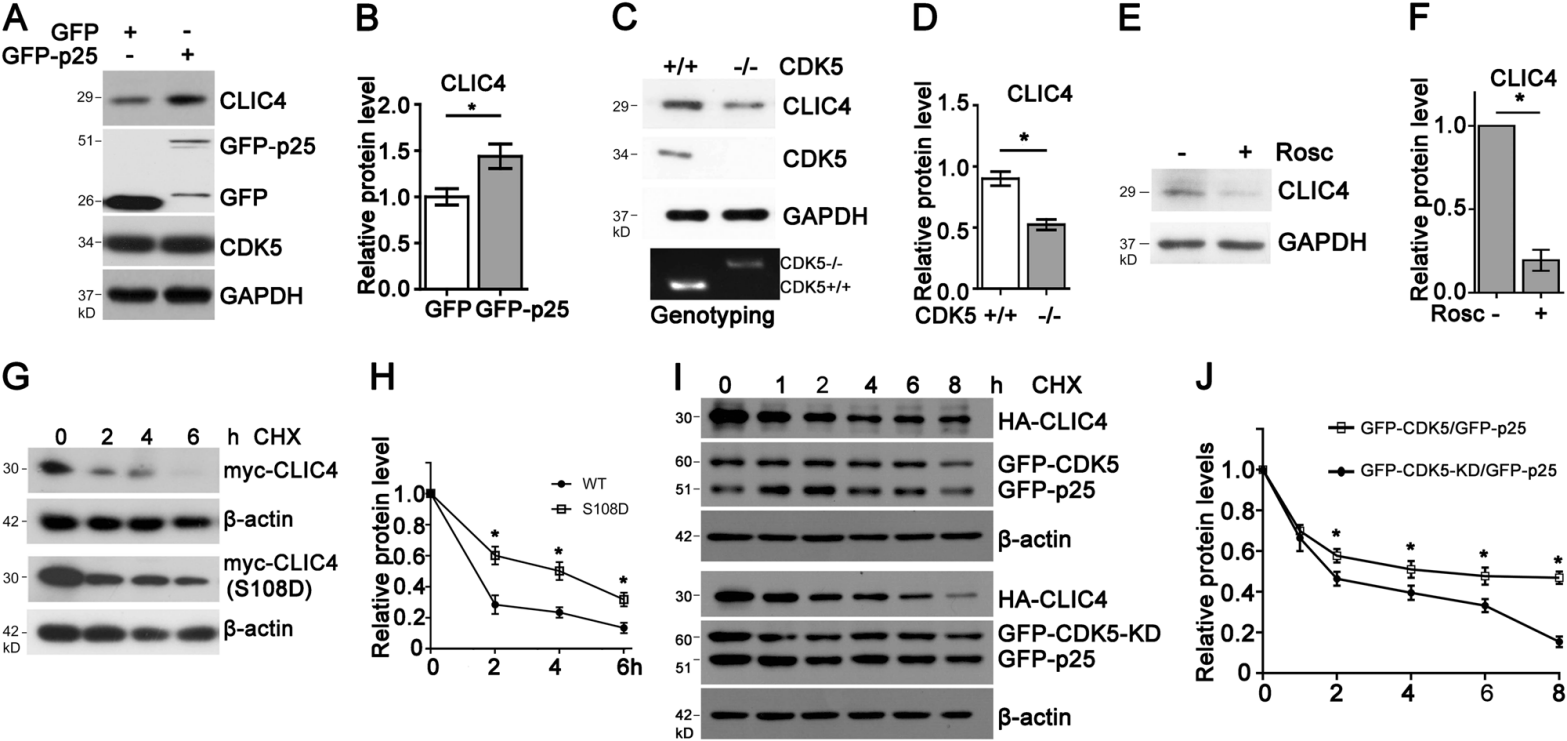
CDK5 promotes CLIC4 protein stability. **a**, **b** Protein levels of CLIC4 in N2a cells transiently transfected with GFP tag or GFP-p25 for 24 h. Relative CLIC4 levels were quantified in **b** (*n* = 3 experiments). **c**, **d** CLIC4 and CDK5 protein levels in the brain homogenates of wild-type and CDK5−/− littermate embryos at E16.5. Embryos were genotyped and brain samples were analyzed by western blotting (**c**). Upper band in the agarose gel indicates CDK5−/− genotype, while lower one means wild-type. Relative CLIC4 levels were quantified in D (*n* = 3 experiments, 4 embryos each group). **e**, **f** Immunoblotting of lysates of primary cortical neurons treated with 5 μM Roscovitine for 24 h. Relative CLIC4 levels were quantified in **f** (*n* = 3 experiments). **g**, **h** Turnover of myc-CLIC4 and myc-CLIC4 (S108D) in N2a cells treated with 100 μM cycloheximide (CHX) for indicated times. Cell lysates were immunoblotted with anti-myc and anti-β-actin antibodies (**g**). Normalized myc-tagged CLIC4 proteins levels were quantified in **h** (*n* = 3 experiments). **i**, **j** Turnover of HA-CLIC4 in N2a cells transiently transfected with HA-CLIC4/GFP-CDK5/GFP-p25 or HA-CLIC4/GFP-CDK5-KD/GFP-p25 and treated with CHX for indicated times. Lysates were subjected to western blotting for HA-tagged and GFP-tagged proteins and β-actin (**i**). Normalized HA-CLIC4 protein levels were quantified in **j** (*n* = 3 experiments). Data are presented as the mean and SEM, and were analyzed by unpaired Student’s *t*-test (**b**, **d**, **f**) or two-way ANOVA test (**h**, **j**). *P < 0.05

As mentioned above, upregulation of CLIC4 and aberrant activation of CDK5 were observed in several neuronal apoptosis models. And, we also identified that CLIC4 acts as a substrate of CDK5. So we wondered whether phosphorylation of CLIC4 by CDK5 is responsible for the accumulation of CLIC4. To test this, GFP-p25 was over-expressed in N2a cells to induce the activation of CDK5, which resulted in enhanced endogenous CLIC4 protein level (Fig. 4a, b). Furthermore, the CLIC4 protein level was about half the amount in CDK5 knockout embryo brain compared to wild-type (Fig. 4c, d). Pharmaceutically inhibition of CDK5 kinase activity by Roscovitine reduced CLCI4 protein level in primary cortical neurons (Fig. 4e, f). Silencing CDK5 expression by transfecting with shRNA targeting CDK5 in N2a cells also led to decreased CLIC4 protein level (Supp. Figure 2).

These results indicate that the protein level of CLIC4 is closely related to CDK5 and its kinase activity. Considering that CDK5 is capable of phosphorylating CLIC4 at serine 108, we wondered whether this phosphorylation mediates protein turnover of CLIC4. To test this, we performed cycloheximide chase assays in N2a cells. We first constructed another CLIC4 mutant CLIC4 (S108D), which mimics the phosphorylation status of CLIC4. We found the turnover of wild-type CLIC4 was much quicker than CLIC4 (S108D) (Fig. 4g, h). Furthermore, CDK5/p25 or kinase-dead^25^ CDK5(CDK5-KD)/p25 were over-expressed with HA-CLIC4 in N2a cells. We observed that the turnover of CLIC4 with CDK5-KD/p25 was also much quicker than with CDK5/p25 (Fig. 4i, j). These results indicate that phosphorylation of CLIC4 by CDK5 promotes its protein stability.

CLIC4 was reported to translocate from cytoplasm to nucleus in certain apoptotic cells^18^. To determine whether serine 108 phosphorylation interferes the sub-localization of CLIC4, primary cortical neurons were transfected with myc-tagged wild-type CLIC4, CLIC4 (S108A), or CLIC4 (S108D) and stained with antibody against myc to visualize their localization. We observed that the phosphorylation status did not affect the subcellular localization of CLIC4. Wild-type CLIC4, CLIC4 (S108A), or CLIC4 (S108D) all primarily localized in cytoplasm (Supp. Figure 3).

### CLIC4 promotes neuronal apoptosis

**Fig. 5:**
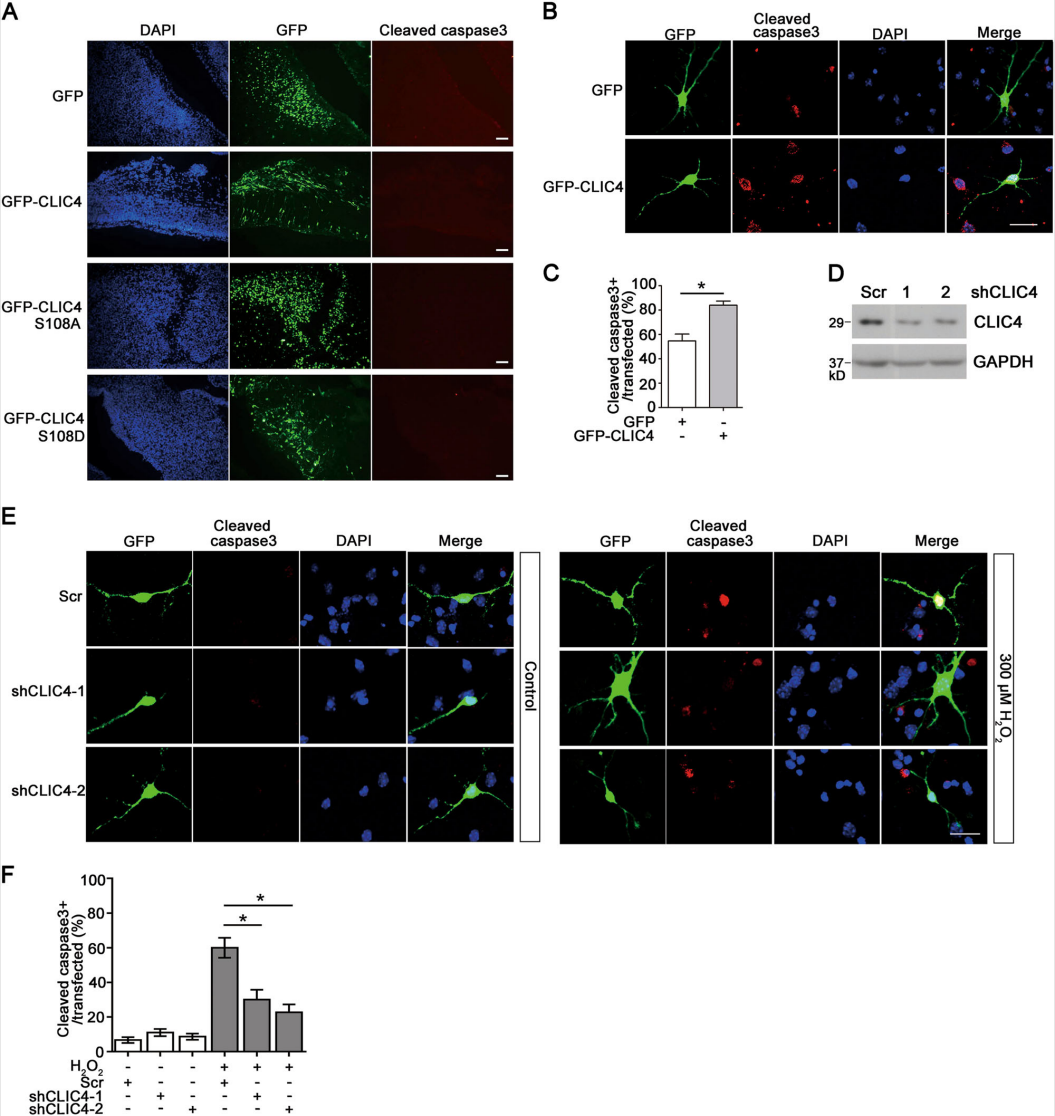
CLIC4 sensitizes neurons to apoptosis. **a** E14.5 embryonic cortical neurons were transfected with GFP, GFP-CLIC4, GFP-CLIC4 (S108A), or GFP-CLIC4 (S108D) usingin utero electroporation. 48 h after transfection, embryonic brains were dissected and fixed. Cortex tissue slices were immunostained with anti-cleaved caspase3 antibody. Scale bar = 100 μm. **b**, **c** Apoptosis of primary neurons transfected with GFP alone or GFP-CLIC4 and treated with 300 μM H_2_O_2_ was measured by cleaved caspase3 immunostaining. The percentages of the cleaved caspase3 positive cells to all transfected cells were calculated (**b**) (over 200 neurons were analyzed in each group, *n* = 3 experiments). Scale bar = 20 μm. **d** Protein levels of CLIC4 in N2a cells transfected with Scramble shRNA, CLIC4 shRNA-1, or CLIC4 shRNA-2. Cells were lysed 72 h later and CLIC4 expression was analyzed by western blotting. **e**, **f** Apoptosis of primary neurons transfected with Scramble shRNA, CLIC4 shRNA-1, or CLIC4 shRNA-2 was measured by cleaved caspase3 immunostaining. Primary cortical neurons were transfected at DIV3. At DIV8, cells were treated with 300 μM H_2_O_2_ or PBS control for 6 h. After fixation, cells were immunostained with cleaved caspase3. Successfully transfected cells also expressed GFP protein encoded by the shRNA plasmids. Apoptosis was measured by the percentage of the cleaved caspase3 positive cells to all transfected cells (over 200 neurons were analyzed in each group, *n* = 3 experiments). Scale bar = 20 μm. Data are presented as the mean and SEM, and were analyzed by unpaired Student’s *t*-test (**c**) or one-way ANOVA test (**f**). *P < 0.05

Considering the fact that CLIC4 protein levels were significantly increased during apoptosis in neurons, we wonder whether overexpressing CLIC4 alone without stresses like H_2_O_2_ could trigger apoptosis in neurons. To test this, GFP, GFP-CLIC4 or its mutants GFP-CLIC4 (S108A) and GFP-CLIC4 (S108D) were expressed in embryonic cortical neurons in vivo by in utero electroporation. Cleaved caspase3 immunostaining revealed no significant apoptosis in either group (Fig. 5a). Then we speculated that overexpression of CLIC4 may promote the apoptosis of neurons with exposure of H_2_O_2_. To verify this hypothesis, primary cortical neurons transfected with GFP tag alone or GFP-CLIC4 were treated with H_2_O_2_. We found that CLIC4 overexpressing neurons exhibited much higher levels of cleaved caspase3 than the GFP overexpressing neurons (Fig. 5b, c). This indicates that neurons possessing high level of CLIC4 were more likely to undergo apoptotic process under oxidative stress. In contrast, silencing CLIC4 by shRNA in primary cortical neurons attenuated the neuronal apoptosis induced by H_2_O_2_ (Fig. 5d–f). These results suggest that CLIC4 makes neurons more vulnerable to stresses like H_2_O_2_.

### CLIC4 inhibitor IAA94 attenuates neuronal death

**Fig. 6:**
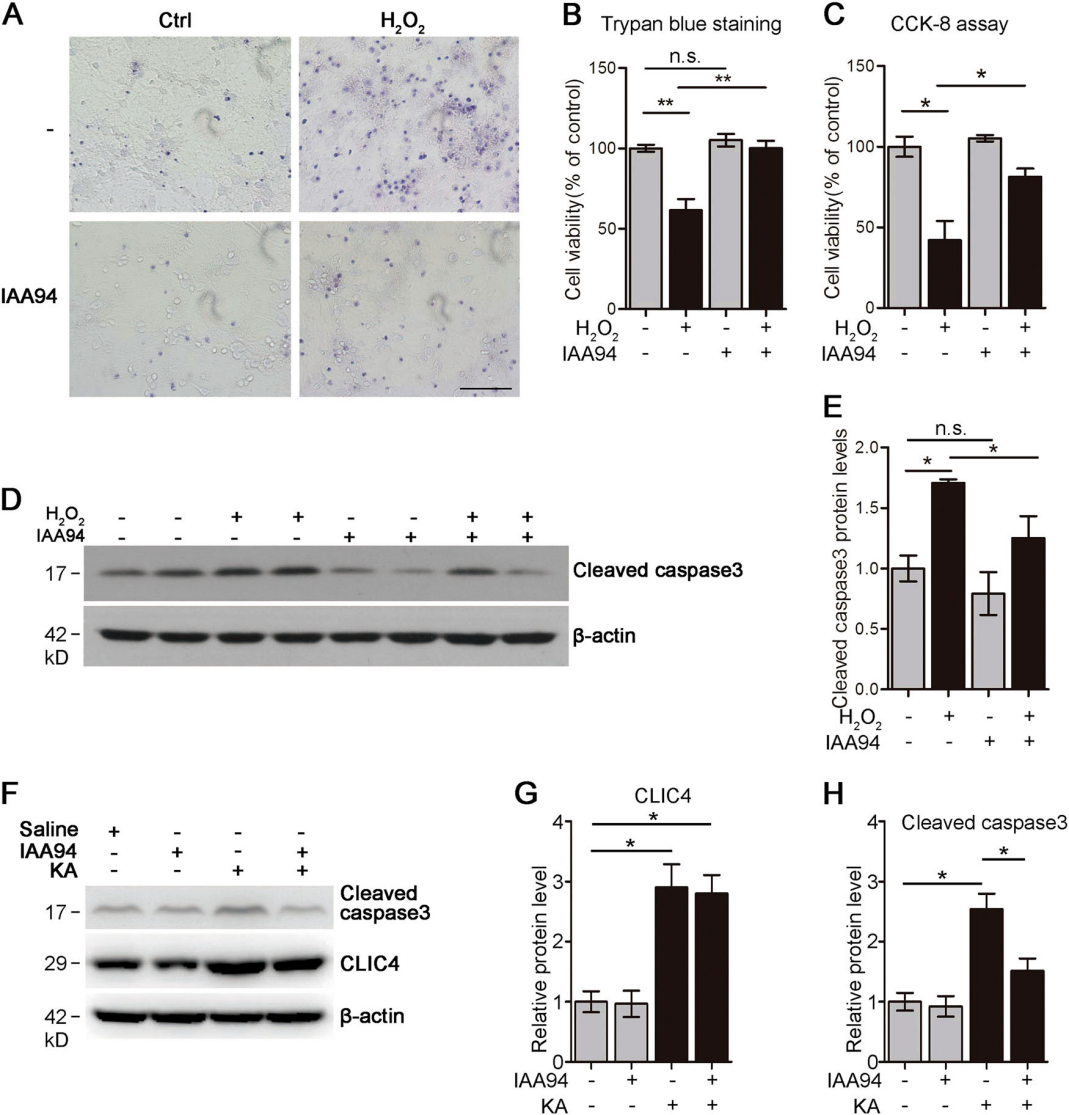
IAA94 prevents neuronal death induced by oxidative stress. **a**–**c** Cell viability of primary cortical neurons was measured by Trypan blue staining and CCK-8. At DIV8, neurons were treated with or without 300 μM H_2_O_2_ for 6 h in the presence or absence of 50 μM IAA94. Then cell viability was determined via Trypan blue staining (by the ratio of the unstained cell number to the total cell number; about 100 cells per coverslip were counted and at least 3 coverslips were used) or CCK-8 assay (*n* = 3 experiments). Scale bar = 100 μm. **d**, **e** Cleaved caspase3 levels in neurons treated with or without 300 μM H_2_O_2_ for 6 h in the presence or absence of 50 μM IAA94. Cell lysates were subjected to immunostaining analysis. Relative cleaved caspase3 levels were quantified in E (*n* = 3 experiments). **f**, **h** Protein levels of CLIC4 and cleaved caspase3 in hippocampi of mice injected with indicated drugs. Adult mice were injected with 10 μl 50 mM IAA94 into lateral ventricle, then 1 h later, 1 μl KA (1 μg/μl) or 1 μl saline was injected into the same location. 24 h after injection, mouse hippocampi were collected and lysed for immunoblotting. Five mice were use in each group. Relative CLIC4 and cleaved caspase3 levels were quantified in **g** and **h** (*n* = 3 experiments). Data are presented as the mean and SEM, and were analyzed by one-way ANOVA test followed by Tukey test. *P < 0.05; **P < 0.01; n.s. not significant

Since overexpression of CLIC4 was capable of inducing neuronal death and knockdown of CLIC4 alleviated neuronal death, we wondered whether pharmacologically blocking ion channel activity of CLIC4 could also attenuate neuronal death induced by H_2_O_2_ or KA. IAA94 is a commonly used CLIC4 ion channel blocker. The cell viability of primary neurons treated with H_2_O_2_ in the presence or absence of IAA94 were measured by Trypan blue staining (Fig. 6a, b) or cell counting kit-8 (CCK-8) assay (Fig. 6c). H_2_O_2_ caused marked cell viability decreasing in neurons, while IAA94 administration significantly alleviated the neuronal injury caused by H_2_O_2_. IAA94 alone administration has no effect on neuronal cell viability. Western blotting results also showed that IAA94 could reduce the level of cleaved caspase3 under H_2_O_2_ treatment (Fig. 6d, e). As demonstrated previously, KA injection-induced hippocampal neuronal death in vivo. Notably, pre-treatment of mice with IAA94 substantially alleviated the neuronal death induced by KA (Fig. 6f–h). IAA94 alone injection didn’t exhibit neuronal toxicity. Together, these results suggest IAA94 rescues neurons from the apoptosis induced by oxidative stress by suppressing channel activity of CLIC4.

In summary, we identified CLIC4 as a new substrate of CDK5. During neuronal apoptosis induced by oxidative stress, phosphorylation of CLIC4 by CDK5 at serine 108 enhances the protein stability of CLIC4, resulting in CLIC4 accumulation in the neurons. CLIC4 accumulation is capable of inducing neuronal death, while CLIC4 blocker IAA94 prevents neuronal death induced by H_2_O_2_.

## Discussion

CDK5 is an atypical cyclin-dependent kinase, which is involved in the process of neuronal apoptosis. Stress conditions such as oxidative stress could aberrantly activate CDK5 via p25 accumulation caused by calpain-mediated p35 cleavage^26^. CDK5 promotes neuronal apoptosis through phosphorylating many substrates which closely related to neuronal death^27^. Pharmaceutically inhibition of CDK5 activity is capable of preventing neuronal death^28^. However, the detailed mechanism underneath the relationship between CDK5 and neuronal death is not fully understood. Here, we identified CLIC4 as a new substrate of CDK5, and confirmed that CLIC4 has an important role in neuronal death.

CLIC4 belongs to CLICs family proteins, which are concerned with a number of important cellular processes, including cell differentiation, apoptosis, migration as well as protein trafficking, but the correlated mechanisms are still not well known. CLICs themselves distinguish from “normal” ion channels in many ways. Apart from the role of ion channel, soluble CLICs (particularly CLIC4) in the cytoplasm and nucleus are also multifunctional through the pathways that we have not known yet. They could be another kind of substrates unknown enzymes based on the fact that a GST-like structure exists in the N-lobe^29^. But in most situations, they are more likely to act as scaffolding proteins. For example, in the TGF-β pathway, CLIC4 helps Schnurri-2 nuclear localization and interacts with Smad2 and Smad3 to protect them from dephosphorylating in the nucleus^13^.

While we reported that CLIC4 contributed to neuronal death mainly due to its role of just being a “chloride channel”, because of blocking its ion channel activity by IAA94 rescued the damage caused by H_2_O_2_ to a large extent. Calcium has a pivotal role in neuronal activity and apoptosis, and calcium transfer through the endoplasmic reticulum or other organelles requires counterion movement, which may be supplied by chloride channels. Blocking chloride intracellular channels could inhibit calcium uptake by the smooth muscle sarcoplasmic reticulum^30^. Similar consequence may also be found in neurons. On the other hand, CLIC4 also locates in the mitochondrial inner membrane. Anion channels on the mitochondrial inner membrane are thought to contribute to mitochondrial volume regulation and oxidative stress-related inner membrane depolarization^31,32^. Chloride channels might also be involved in the fusion and fission of mitochondria^33^. Nevertheless, the roles of CLIC4 beyond chloride channel are also worthy to be considered in the process of neuronal apoptosis.

Considering the vital role of CDK5 and potential involvement of CLIC4 in neuronal death, we explored whether CLIC4 participates in the neuronal apoptosis induced by CDK5 aberrant activation. We identified that CLIC4 was phosphorylated by CDK5 at serine 108 site (Fig. 2). Serine 108 is located in the join-loop of N-lobe and C-lobe of CLIC4, which is believed to be important for CLIC4 stability^11^. A mutation in this region of CLIC2 identified in patients with intellectual disability was also predicted to impair CLIC2 stability and dynamics^34^. Importantly, aberrant activation of CDK5 in several stress conditions such as H_2_O_2_, glutamate, KA treatment or KCl withdrawal was paralleled with the upregulation of CLIC4 protein level in neurons, indicating that phosphorylation of CLIC4 by CDK5 may enhance the protein stability of CLIC4 (Fig. 3). If CDK5 activity is inhibited either by pharmaceutical approach (Roscovitine) or genetic reduction, CLIC4 level will decrease significantly (Fig. 4). What’s more, the degradation rate of CLIC4 (S108D) is much slower relative to that of wild-type CLIC4, suggesting that phosphorylation of serine 108 has an important role in CDK5-mediated posttranslational regulation of CLIC4.

Inasmuch as CDK5 also has critical roles in neuronal migration and differentiation, phosphorylation of CLIC4 by CDK5 during brain development could be a contributor to this process. We have found that CLIC4 level reached the peak during mouse embryonic stage in the brain and was downregulated after birth (data not shown). It was also reported that CLIC4 formed complex with actin, tubulin, and 14-3-3 isoforms^12^. CLIC4 was also reported to be involved in regulation of RhoA activity^35^. Another CLICs family member CLIC1 co-localized with RhoA^36^ and CLIC1 promoted neurite elongation in retinal ganglion cells^37^. All these evidences strongly suggest that CLIC4 may also participate in neuronal migration and differentiation.

In conclusion, we found that CLIC4 protein level was increased during neuronal apoptosis, and this might be due to the phosphorylation of CLIC4 by CDK5/p25. Even though the mechanism of CLIC4 mediates neuronal death is still unknown, knockdown of CLIC4 or blocking CLIC4 channel activity by IAA94 rescued neuronal death caused by H_2_O_2_ or KA. These results suggested that CLIC4, acting as a substrate of CDK5, mediates neuronal death. Targeting CLIC4 may therefore provide a therapeutic approach for neuronal death mediated by CKD5 aberrant activation.

## Electronic supplementary material

supplementary figures
