# Supplementary material for: Cyclin-dependent kinase 5-mediated phosphorylation of chloride intracellular channel 4 promotes oxidative stress-induced neuronal death
Source: Cell Death Dis. 2018 Sep 20;9(10):951. doi: 10.1038/s41419-018-0983-1 (PMC6147799; doi:10.1038/s41419-018-0983-1)
Supplement: Supplementary file 1 — supplementary figures [file 41419_2018_983_MOESM1_ESM.pdf]

Supplementary Figures

Cyclin-dependent kinase 5–mediated phosphorylation of chloride intracellular channel 4 promotes oxidative stress–induced neuronal death

Dong Guo, Wenting Xie, Pan Xiong, Huifang Li, Siqi Wang, Guimiao Chen, Yuehong Gao, Jiechao Zhou, Ye Zhang, Guojun Bu, Maoqiang Xue, Jie Zhang

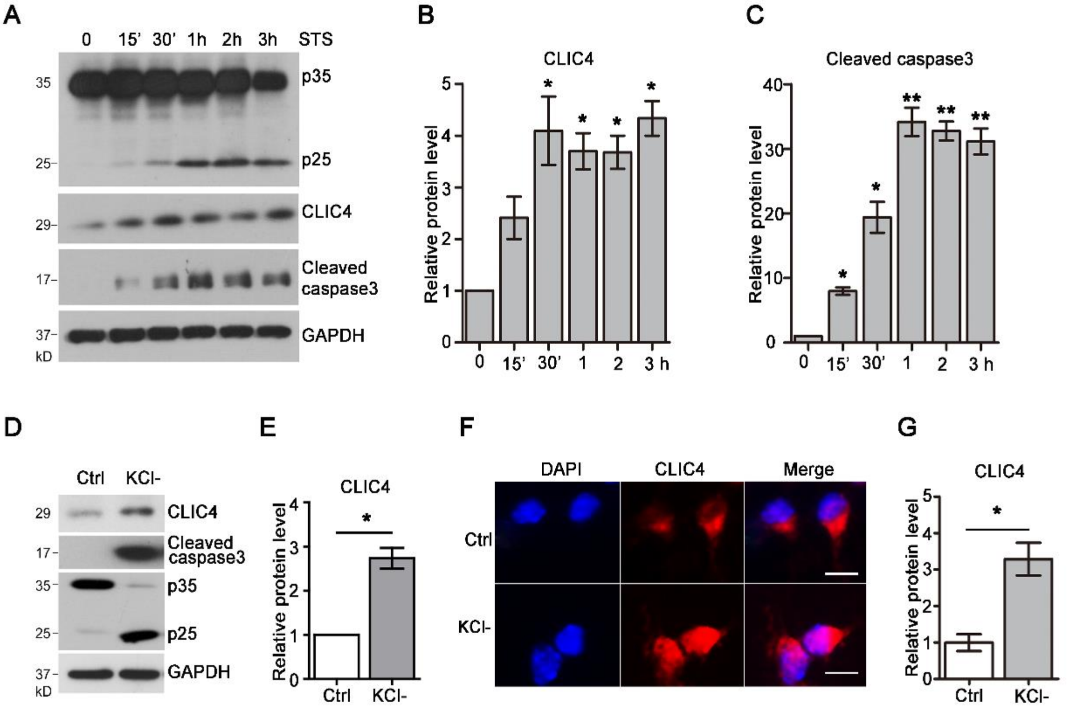

Supplementary Figure 1. STS treatment and KCl withdraw induced neuronal death and upregulation of CLIC4 protein. A-C, Protein levels of CLIC4, p35/p25 and cleaved caspase3 in primary cortical neurons (DIV8) treated with 1 μM STS for indicated times. Relative expression levels of CLIC4 and cleaved caspase3 were quantified in B and C (n = 3 experiments). **D-E**, Protein levels of CLIC4, p35/p25 and cleaved caspase3 in CGNs with or without KCl withdrawal treatment. At DIV4, culture medium of CGNs containing 30 mM KCl was replaced with medium containing 5 mM KCl and maintained for 12 h. Relative CLIC4 levels were quantified in E (n = 3 experiments). **F-G**, Immunostaining of CLIC4 in CGNs with or without KCl withdrawal treatment. DAPI was used as a nucleus dye. The relative intensity of CLIC4 in each cell was qualified in O (n = 3 experiments, over 20 cells were qualified in each experiment). Scale bar = 20 μm. Data are presented as the mean and SEM, and were analysed by one-way ANOVA test followed by Dunnett test (B, C) or unpaired Student's *t* test (E, G). *, $P<0.05$; **, $P<0.01$.

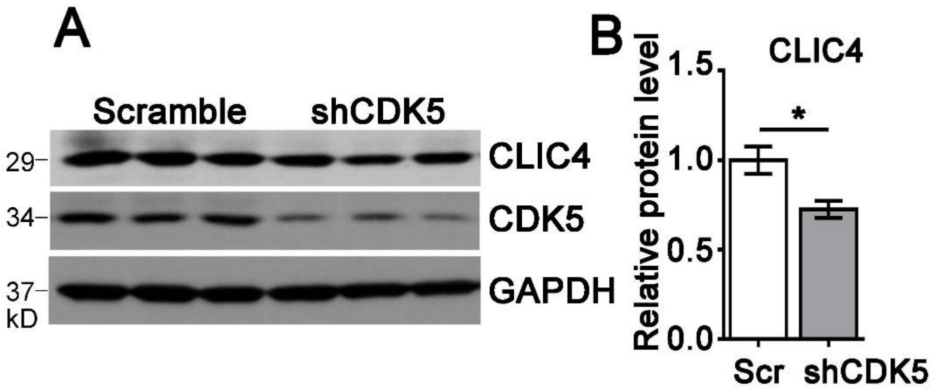

Supplementary Figure 2. Knock-down of CDK5 decreased endogenous CLIC4 protein level. A-B, Expression levels of CLIC4 and CDK5 proteins in N2a cells transiently transfected with scramble shRNA or shCDK5 plasmids. 72 h after transfection, cells were lysed to do the Western blotting of indicated proteins. Relative CLIC4 protein levels were quantified in B (n = 3 experiments). Data are presented as the mean and SEM, and were analysed by unpaired Student's *t* test. *, $P<0.05$.

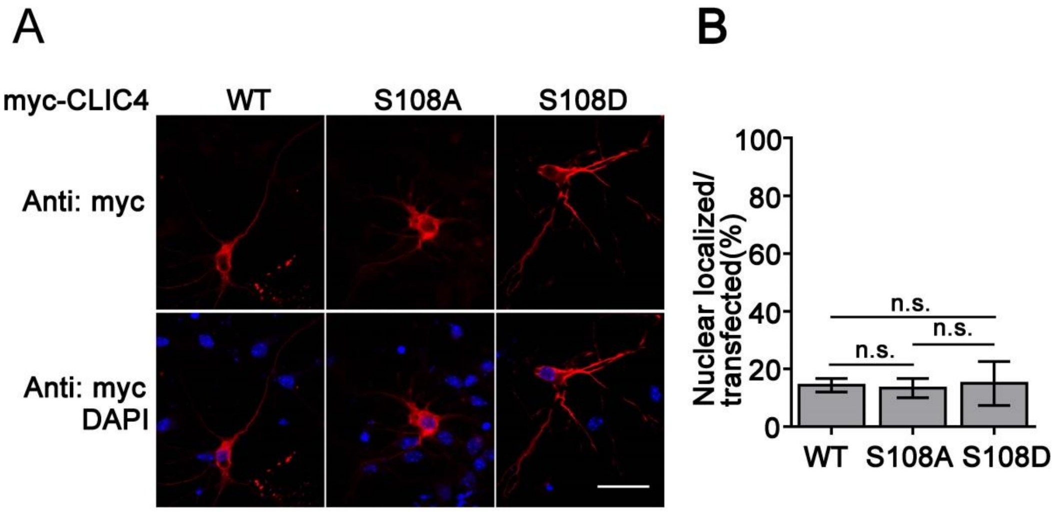

Supplementary Figure 3. Phosphorylation of CLIC4 by CDK5 does not affect its subcellular localization. A-B, Immunostaining of primary cortical neurons transfected with myc-CLIC4, myc-CLIC4 (S108A) or myc-CLIC4 (S108D). Cells were fixed 24 h after transfection and stained with anti-myc antibody. The percentages of nuclear localized myc-CLIC4 or mutants in all transfected cells were calculated (B). Over 100 cells and at least 3 coverslips were counted in each group (n = 3 experiments). Scale bar = 20 μm. Data are presented as the mean and SEM, and were analysed by one-way ANOVA test followed by Tukey test. n.s., not significant.
